# Emergency Repair of an Isolated Traumatic Avulsion of the Right Main Stem Bronchus in a 7-Year-Old Girl

**DOI:** 10.1055/s-0039-1681038

**Published:** 2019-05-26

**Authors:** Tatjana Tamara König, Eva Wittenmeier, Oliver J. Muensterer

**Affiliations:** 1 Department of Pediatric Surgery, University Medicine Mainz, Mainz, Germany; 2 Department of Anesthesiology, University Medicine Mainz, Mainz, Germany

**Keywords:** bronchial avulsion, tension pneumothorax, pediatric trauma, blunt thoracic trauma

## Abstract

**Introduction** Isolated tracheobronchial injury after blunt trauma of the chest is rare. Because of the high elasticity of the chest in children, they occur mainly in the pediatric population.

**Case Report** We report a case of a 7-year-old girl who experienced complete avulsion of the right main bronchus at the level of the carina after a horse-riding accident. The patient presented with extensive emphysema of the upper chest, neck, and face and severe respiratory distress. Endotracheal intubation led to tension pneumothorax. After insertion of two 17-mm thoracostomy tubes, pneumothorax and a massive air leak persisted. Isolated central bronchial injury was confirmed by computed tomography of the chest. Bronchoscopically guided selective intubation of the left main stem bronchus failed and the patient desaturated, requiring immediate salvage right posterolateral thoracotomy. Simultaneous occlusion of the defect, stabilization, and subsequent selective left lung intubation was possible only after placing a suture at the tracheal rim of the defect for retraction allowing compression of the defect and keeping the lumen open at the same time.

**Conclusion** A cluster of clinical signs with subcutaneous emphysema and refractory pneumothorax with air leak of the thoracotomy tube is indicative of bronchial injury. Endotracheal intubation should be postponed in these cases until after thoracostomy tube placement, if possible. Placing a retraction suture during repair is a maneuver that helps to occlude the defect and keep the remaining tracheobronchial lumen open at the same time to establish crucial ventilation of the contralateral lung.

## Introduction

Thoracic trauma is a rare condition, seen in only 13% of pediatric trauma patients. More than 80% of the affected patients suffer from severe combined injuries after road traffic injuries or a fall. 1 Pulmonary contusion or rib fractures are the most common findings in the pediatric population (50% of cases), followed by pneumothorax in around 37% of patients. 1 In contrast, traumatic tracheobronchial injury is exceptionally rare, present in only 0.05 to 3% of cases. 1 2 Clinical signs vary or may be misleading, 1 making these injuries hard to diagnose. Traumatic airway disruption is a potentially lethal condition with mortality up to 30%, with one half of these deaths occurring during the first hour after trauma. 2 3

Injury patterns after blunt thoracic trauma in children differ dramatically from those of adults. The high elasticity of the thoracic wall in children transmits the impact of external force on the intrathoracic and mediastinal organs with little external signs of injury. 1 3 Associated rib fractures, for instance, are only seen in 25% of tracheobronchial injuries. 2 Also, tension pneumothorax is three times more frequent in children compared with adults, due to increased mobility of the mediastinum. 1

In most cases of lower airway injuries, a simple bronchial repair is successful. 4 Tension-free anastomosis should be completed with interrupted sutures, tying the knots outside the lumen. 2 More severe or combined injuries have been repaired successfully on cardiopulmonary bypass. 5 Short-term complications include persistent bronchopleural fistulas and mediastinitis. 2 In due course, anastomotic stenosis can develop, so that follow-up bronchoscopy is recommended for symptomatic patients. In such cases, conservative management with endoscopic dilatations is possible. 4

## Case Report

**Fig. 1:**
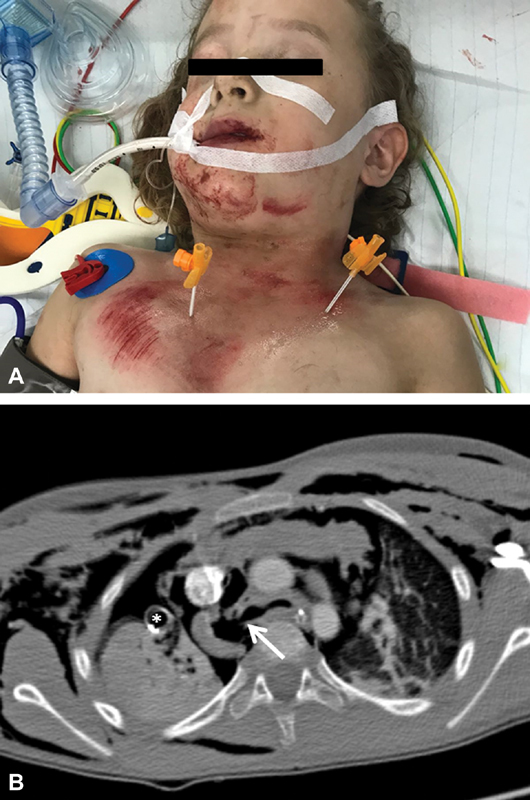
( **A** ) Clinical appearance of the patient in the trauma bay after intubation and bilateral needle thoracostomy; ( **B** ) computed tomography imaging displaying rupture of the right main stem bronchus (arrow), massive soft tissue and mediastinal emphysema, and significant right pneumothorax and in spite of drainage (*thoracostomy tube).

A 7-year-old girl presented to our emergency department after horse riding accident. Although the event was unwitnessed, most likely the patient fell off the horse and was stepped onto the chest by one of the hind hoofs. At arrival in the trauma bay, she was placed on a stretcher in prone position, chest propped up on the arms, displaying signs of severe dyspnea with tachypnea, increased work of breathing, and both inspiratory and expiratory stridor. Her oxygen saturation (SaO _2_ ) was stable around 90% after application of supplemental oxygen. The patient was fully aware and conscious (Glasgow Coma Scale 15), but agitated. On physical examination, there were bruises on the throat and right upper chest with massive subcutaneous emphysema of the upper chest, neck, and face. The patient was given sedatives, leading to desaturation. Still, there was no obvious difference in breath sounds on both sides, so bilateral needle thoracostomy was performed, and the patient was endotracheally intubated ( Fig. 1A ). After intubation and positive-pressure ventilation, oxygen saturation and blood pressure dropped. At this time, right tension pneumothorax was diagnosed, and two 17-mm thoracostomy tubes were placed.

### Diagnostic Assessment

A massive air leak was appreciated from the chest tubes ( Video 1 ). The SaO _2_ improved to 84%. Computed tomography (CT) scan showed a complete avulsion of the right main stem bronchus at the level of the carina, a residual pneumothorax with mediastinal shifting, atelectasis of the right lung, as well as emphysema of the mediastinum and soft tissue ( Fig. 1B ). The only skeletal thoracic injury was a fracture of the contralateral left first rib. The blood gas showed acidosis (pH 7.16), and hypercapnia (partial pressure of carbon dioxide 63 mm Hg).

**Video 1**

Massive air leak after application of negative pressure on the chest tubes.

### Therapeutic Intervention

The patient was taken to the operating room immediately. With positive-pressure ventilation, oxygen saturation dropped continuously in spite of two working thoracostomy tubes and manual high frequency, low tidal ventilation. Single lung ventilation of the contralateral lung was our primary approach for ventilation during thoracotomy. An attempt of bronchoscopy-guided selective left main stem intubation failed because of impaired visualization due to blood in the airway. During bronchoscopy, the patient desaturated (SaO _2_ <10% for 5 minutes, heart rate dropped to 60/min).

### Intraoperative Course

**Fig. 2:**
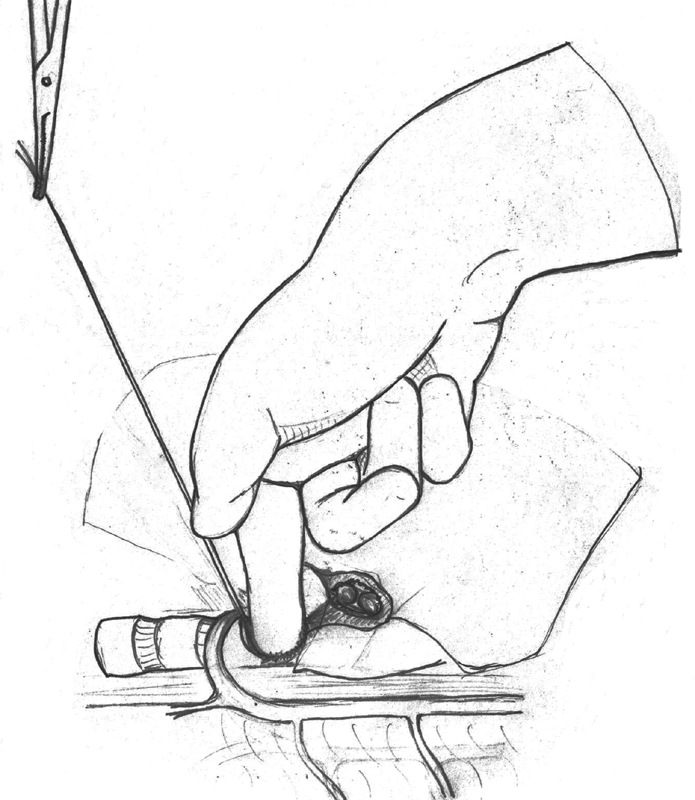
Intraoperative placement of traction suture at the caudal tracheal rim of the defect, allowing simultaneous occlusion of the defect and patency of the left main stem bronchus.

The patient was placed in left lateral decubitus position and immediate, salvage posterolateral thoracotomy was performed. The defect was identified at the level of the carina, leaving no proximal bronchial stump to place a clamp. Temporary occlusion of the defect with the surgeons' finger allowed some ventilation of the left lung to gradually improve the oxygen saturation. Placing the finger on the defect, however, partially occluded the left main bronchus at the same time, prohibiting selective intubation and sufficient ventilation of the left lung. We decided to place a 3-0 polypropylene suture for retraction at the caudal rim of the tracheal defect, which allowed simultaneous occlusion of the defect and patency of the left main stem bronchus. Hence, intubation under visual and tactile guidance with a preloaded 5.0 tube, using bronchoscope as “guidewire,” was possible ( Fig. 2 ). After cardiorespiratory stabilization, the reconstruction of the bronchus was performed. The anastomosis was completed with a posterior running 3-0 polypropylene. The anterior wall was closed with interrupted sutures knotted outside the lumen. There was no tension on the anastomosis and no air leak at under-water testing. A single chest tubes was left in place postoperatively.

### Postoperative Course

After surgery, pressure-controlled ventilation was continued on the intensive care unit. Broad-spectrum antibiotics were administered. Body temperature was lowered to 35°C over a period of 48 hours for neuroprotection. On the second postoperative day, bronchoscopy was performed showing an intact anastomosis, so that the breathing tube was retracted into the trachea 2 cm above the level of the carina. The patient was extubated on postoperative day 7 and discharged from the hospital on postoperative day 24 after weaning of sedatives and antimicrobial treatment of colonization of the trachea with *Enterobacter cloacae* according to our microbiologists' protocol. On physical examination, an alar scapula persisted. Neurologic exam was unremarkable.

## Discussion

Severe thoracic injury after trauma is rare in young children, but may lead to significant morbidity or even death within a short period of time after trauma. After thoracic trauma, clinical signs like subcutaneous emphysema and massive air leak in the thoracostomy tube are indicative of tracheobronchial disruption. These symptoms are infrequently encountered by clinicians, which may lead to delayed recognition and treatment.

Intubation of our patient before placing a chest tube led to life-threatening decompensation. In a patient with suspected bronchial injury, positive-pressure ventilation should be avoided whenever possible because it may exacerbate the pneumothorax. When a single chest tube does not provide adequate decompression of a pneumothorax, a second chest tube should be placed. A large, ongoing air leak in combination with progressive subcutaneous emphysema is highly suspicious of relevant airway injury. Clinicians need to be aware of this cluster of symptoms.

In most cases, plain chest radiograph is recommended for pediatric patients with thoracic trauma, revealing relevant fractures, pneumothorax, and mediastinal pathologies that need to be addressed immediately. 1 There is no consensus about the primary use of chest CT in this group. Even though CT scan might have a higher sensitivity for pathologies like lung contusion or a small pneumothorax, these additional findings rarely lead to a change in management. 1 6 The cancer risk after thoracic CT in childhood is estimated as 25/10,000 for girls and 7.5/10, 000 for boys. 7 Therefore, some authors argue that CT of the thorax should be considered only in patients with pathological findings on plain film X-ray. 6 In our particular case, a primary CT was obtained after stabilization of the patient, which was helpful in locating the injury preoperatively.

Intraoperatively, clamping of the bronchial avulsion was impossible due to the proximal injury at the carina. Temporary occlusion of the tracheal defect was possible, but partially occluded the remaining intact left bronchus at the same time. Only after placement of a traction suture at the rim of the defect, it was possible to manually direct the tube into the left bronchus and secure ventilation. Inability to clamp the defect must be anticipated in situations like these, since 80% of tracheobronchial injuries occur within 2.5 cm of the tracheal bifurcation by entrapment of the airway between the sternum and the vertebral column. 2 Therefore, our technique employing a traction stitch may be helpful and life-saving in similar cases.

Thoracotomy and primary anastomosis without further measures are the main surgical approach for tracheobronchial injury in children 2 and adults. 8 Both running or interrupted sutures are used, according to the literature. 2 8 9 Risk factors for anastomotic leakage include inadequate suture pitches, discrepancy of the bronchial diameter, high tension on the anastomosis, and poor visualization of the operative field. 9 Intercostal muscle flap, 8 suture holders, 9 or bronchial plication of the membranous portion of the bronchus in cases of discrepancy of the bronchial diameter 10 may be used to reduce the risk of complication in a stable patient.

## Conclusion

Even though tracheobronchial injuries in children are extremely rare, they require urgent intervention. The combination of air leak after thoracostomy and subcutaneous emphysema should be considered bronchial injury until proven otherwise. In these cases, intubation may lead to respiratory deterioration and should be postponed in a patient with sufficient respiratory compensation until a chest tube is in place and the patient is in a controlled environment, such as the operating room, where thoracotomy can be performed immediately. Finally, our technique of placing a retraction suture on the upper rim of the defect allows the surgeon to temporarily occlude the defect with a finger while at the same time facilitating ventilation of the contralateral lung.

## Notes

New Insights and the Importance for the Pediatric Surgeon The combination of air leak after thoracostomy and subcutaneous emphysema should be considered bronchial injury until proven otherwise. Our technique of placing a retraction suture on the upper rim of the defect allows the surgeon to temporarily occlude the defect with a finger while, at the same time, facilitating ventilation of the contralateral lung.
